# An isolated duodenal perforation in pediatric blunt abdominal trauma: a rare but distinct possibility

**DOI:** 10.1186/s41038-015-0008-6

**Published:** 2015-06-25

**Authors:** Anjan Kumar Dhua, Manoj Joshi

**Affiliations:** Department of Pediatric Surgery, Pondicherry Institute of Medical Sciences, Quarter E7, PIMS Hospital Campus, Pondicherry, 605 014 India

**Keywords:** Blunt trauma abdomen, Duodenorrhaphy, Isolated duodenal perforation, Pediatric trauma

## Abstract

Isolated duodenal perforation (IDP) in pediatric trauma is rarely reported. Since most of the children with blunt trauma are managed expectantly, timely diagnosis is imperative to avoid morbidity and mortality. We report a case of IDP and emphasize on certain specific clinical features indicating possibility of duodenal injury. We also stress upon the role of early contrast-enhanced computerized tomography (CECT) in such cases.

## Background

Duodenal injuries are rare in pediatric population, and isolated duodenal perforation (IDP) is even rarely reported. Overall, duodenal injuries accounts for only 3–5 % of all trauma cases [1]. Exact incidence of IDP in children is not known due to its rarity. Isolated involvement of duodenum is rare because of its deep retroperitoneal location. More commonly, other surrounding organs are concomitantly injured in the high energy transfers involved in the trauma. Clinical detection of IDP therefore is a challenge to the surgeon because of the absence of florid signs.

## Case presentation

A 7-year-old boy was brought to the emergency room with history of crush injury by bullock cart 1 h before presentation. The boy was complaining of severe pain in the upper abdomen and had two episodes of non-bilious vomiting. On examination, pulse rate was 130/min and BP was 110/70 mmHg. The abdomen was tender over the right hypochondrium and lumbar region. The right lower limb was flexed at the hip joint, and passive extension was painful. There was no pallor. An abdominal radiograph was nonspecific and without evidence of free air. Abdominal ultrasound demonstrated minimal fluid in the pelvis and unremarkable solid organs. In view of the hemodynamic stability and unavailability of expert radiologist, the child was initially managed expectantly. The investigations revealed—hemoglobin 120 g/dl, hematocrit 32 %, total leukocyte count 26 × 10^3^/μl, and serum amylase 30 U/L.

He had few episodes of bilious vomiting in the next 12 h. The patient complained of increased abdominal pain and had local abdominal tenderness despite adequate bowel rest. Although the child was hemodynamically stable, he had fever spikes with temperature reaching up to 38.9 °C.

**Fig. 1:**
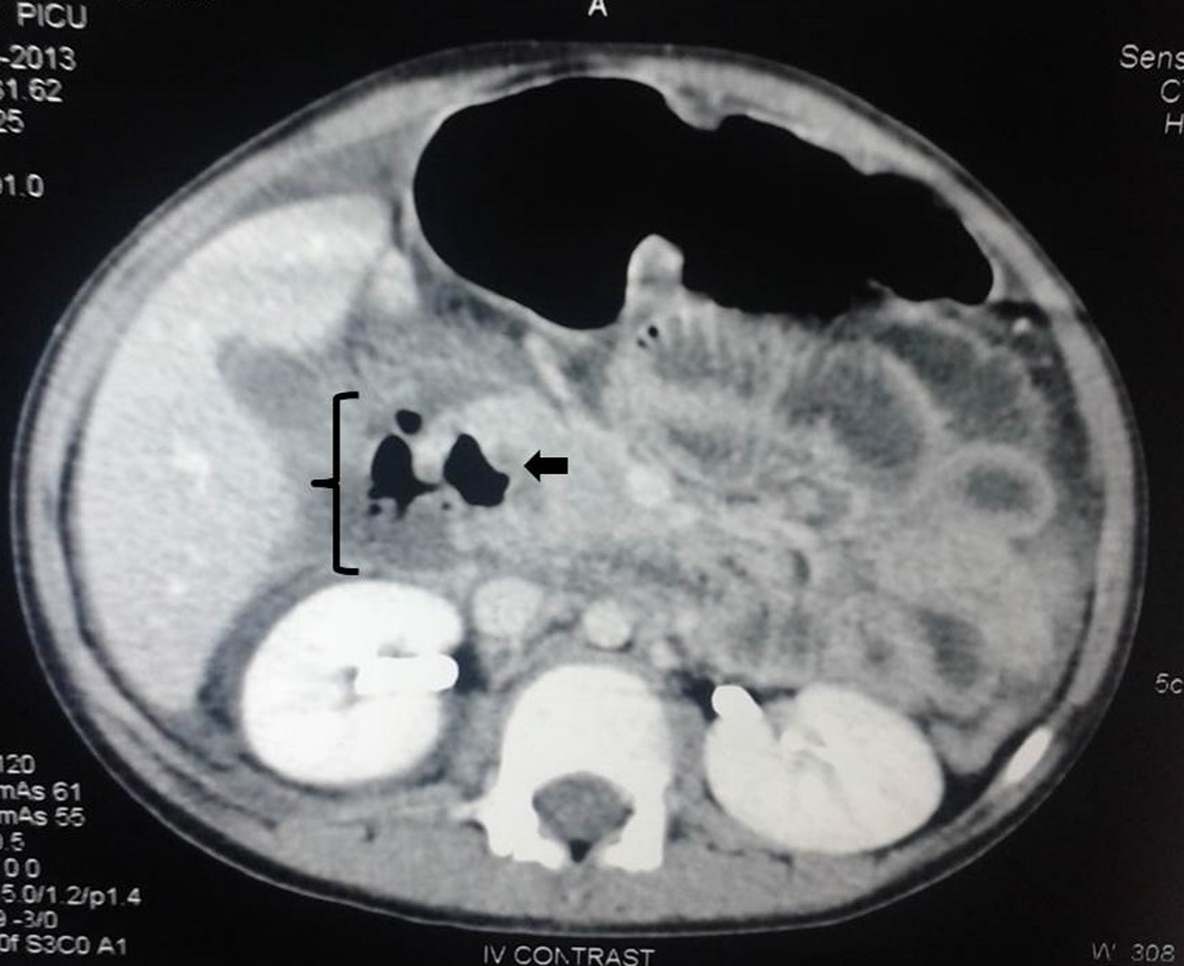
Axial section of contrast-enhanced computerized tomography (CECT) abdomen at the level of D2 (second part of the duodenum, *arrow*) showing discontinuity in the lateral wall of duodenum, extravasation of negative contrast with intraluminal communication, and few air pockets (*brace*)

**Fig. 2:**
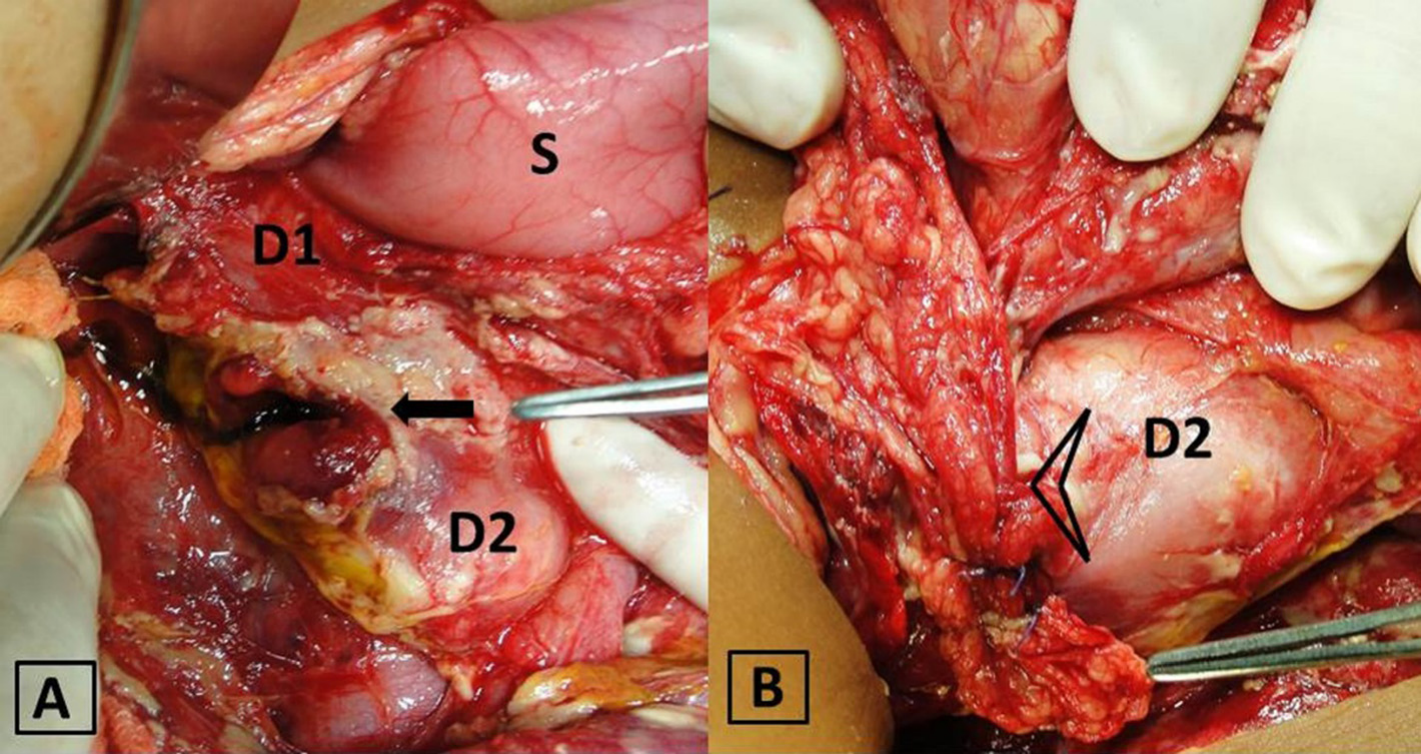
**a** Intra-operative image showing the perforation (*arrow*) in relation to stomach (*S*), first part of the duodenum (*D1*) and second part of duodenum (*D2*). **b** Same region after repair is complete and buttressed by an omental patch (*arrow head*)

A contrast-enhanced computerized tomography (CECT) of the abdomen was done, which revealed free fluid of high density in the peritoneal cavity around the hepato-renal pouch and localized free air in retroperitoneum around second part of duodenum, which was communicating with the lumen (Fig. 1). An exploratory laparotomy was subsequently performed that revealed copious frank pus in the peritoneal cavity. The hepatic flexure was inflamed and revealed numerous flimsy inter-bowel adhesions. After reflecting the cecum and ascending colon medially, the duodenum was identified and was noted to be covered with bile-stained slough. After kocherization, a 2 × 2 cm perforation was seen on the lateral wall of the second part of duodenum (Fig. 2a).

Since the defect was less than 50 % of the circumference, duodenorrhaphy was performed by closing the perforation in a transverse fashion. A pedicled omental patch was added (Fig. 2b). The patient was kept nil per oral for 10 days with supplemental parenteral nutrition and had an uneventful recovery.

### Discussion

IDP in pediatric trauma is scarcely reported. Only isolated cases of IDP following blunt abdominal trauma exists in the English literature [2–4]. DuBose et al. in 2008 reported a series of five adult cases of IDP, which was the largest series thus far in the literature [5]. Concomitant injuries are more common, and overall outcome depends on the nature and the severity of these injuries.

Road traffic accidents are the most common mode of blunt injury to the duodenum [6]. In addition to this, other peculiar mechanisms commonly encountered in children are falls, bicycle handlebar injuries, child abuse, and playground accidents [7–9]. IDP following blunt trauma abdomen (BTA) may occur as a result of “crush injury” or “distraction injury”. The duodenum may get crushed between spine and other hard objects like handlebar or a steering wheel [10]. In our case, the duodenum got crushed between the spine and heavy bullock cart. “Distraction” injuries with perforation occur at the junction of the first and second part of the duodenum [10]. This usually follows sudden deceleration as after high-speed motor vehicle accidents.

Presentation in IDP may be nonspecific initially. Specific signs of perforation may appear late. Symptoms may be mild at the outset and range from mild to severe upper abdominal pain with recurrent vomiting. There are reports of patients with milder symptoms having been discharged from the emergency department only to be readmitted a few hours later as the symptoms worsened [11, 12]. An in-depth review of the literature on IDP showed vomiting and abdominal pain, localized to the right upper abdomen, being the most prevalent symptom [13]. Leukocyte count has been found to be elevated in almost all cases [3, 12, 13]. Serum amylase levels may be raised but is less specific [11, 12]. The triad of vomiting, upper abdominal pain, and leukocytosis, though individually less specific, when present together in BTA, may suggest duodenal injury [13]. Our patient had all these symptoms, which gradually progressed. In addition, he also had painful flexion attitude of the right hip, which may had been related to spasm of right psoas muscle secondary to irritation from the surrounding duodenal fluid. This finding of “psoas spasm” in blunt abdominal trauma was noted in our case and has not been described in literature earlier. Therefore, if consistently present, along with the aforementioned triad, it may clinically suggest duodenal injury.

Abdominal radiograph and sonography may not be useful in diagnosis of IDP due to its retroperitoneal location [14]. The role of early CECT abdomen with oral contrast at this point is crucial. When multi-detector CT is used, sensitivity of 88–93 % can be achieved for detecting bowel injuries in patients with blunt trauma [15]. However, such sensitivity and specificity data of CT in diagnosis of isolated duodenal injuries do not exist in current literature due to its uncommon occurrence [13]. Duodenal perforation is suggested if there is a retroperitoneal collection of contrast medium, extra-luminal gas, or a lack of continuity of the duodenal wall [13]. Since majority of blunt trauma cases in children with stable hemodynamics are managed expectantly, clinical correlation with mentioned CECT features can help surgeons in early decision-making. We retrospectively correlated the clinical symptoms and learned that an early CECT would have helped us in early exploration of this case.

In most of the cases where the perforation is less than 50 % of the circumference, simple duodenorrhaphy is adequate [7]. In addition to primary repair, feeding jejunostomy or a gastrojejunostomy may be added to safeguard the repair [5]. For perforation sizes that preclude a primary repair, techniques like jejunal serosal patches and pedicled mucosal flap with jejunal or gastric island flap have been described in experimental setting with minimal impact in actual clinical setting [10]. Another technique with Roux-en-Y duodenojejunostomy has been described with encouraging results in clinical setting as well [16]. Irrespective of the technique used, the outcome however also depends on the timing of intervention. It has been noted that in duodenal perforation with concomitant injuries, a delay of more than 24 h has a poor outcome [7]. Thus, early recognition of this rare injury is necessitated for a better outcome.

## Conclusions

To conclude, in view of its rarity, a high index of suspicion is necessary to diagnose IDP in children. Since most of the children with BTA are managed conservatively, presence of clinical indicators like upper abdominal pain, vomiting, and raised leukocyte count should prompt an early CECT of the abdomen. “Psoas spasm”, when present, should always alert the surgeon.

## Consent

Written informed consent was obtained from the patient’s parents for the publication of this report and the accompanying images.

## Abbreviations

IDP: isolated duodenal perforation
CECT: contrast-enhanced computerized tomography
IDP: isolated duodenal perforation
BTA: blunt trauma abdomen
